# Supplementary material for: Transcriptome Analysis Revealed the Dynamic and Rapid Transcriptional Reprogramming Involved in Cold Stress and Related Core Genes in the Rice Seedling Stage
Source: Int J Mol Sci. 2023 Jan 18;24(3):1914. doi: 10.3390/ijms24031914 (PMC9916315; doi:10.3390/ijms24031914)
Supplement: Supplementary file 1 [file ijms-24-01914-s001.zip › supplement files.pdf]

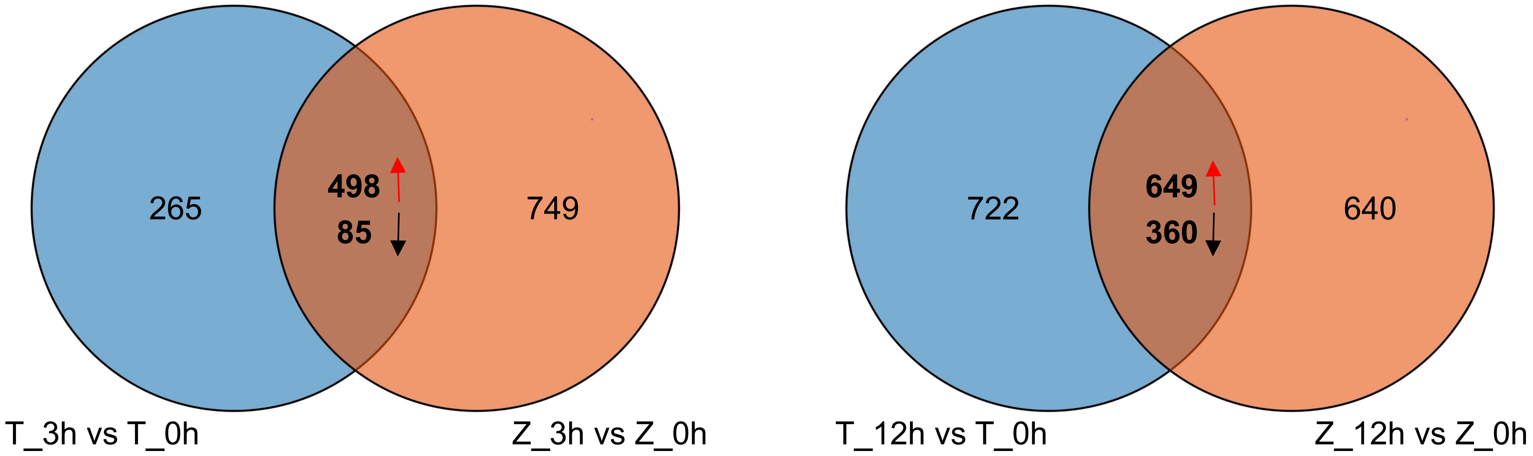

Figure S1. Venn diagram of DEGs induced by cold stress in Towada and ZL31 at the two time points compared with 0 h cold treatment. Up- and downregulated DEGs are indicated by upward- and downward-pointing arrows, respectively.

Table S1. Putative *cis*-regulatory elements of *OsWRKY24* identified in variant regions of the Towada and ZL31 promoters.

**Variant region (Towada)**	**Putative *cis*-element ID**	**Putative function**
-1139 to -1136 bp	GTGA motif	Modulate expression of the tobacco late pollen gene
-572 to -567 bp	AMYBOX2	Conserved sequence found in 5' upstream region of *alpha-amylase* gene
	MYB	Core motif of MYB binding site
-213 to -207 bp	SEF3MOTIFGM	SEF3 binding site found in the 5' upstream region of beta-conglycinin

Online software analysis by PLACE (http://www.dna.affrc.go.jp/PLACE/).

Table S2. Primers for qRT-PCR.

Gene	Forward primer (5'-3')	Reverse primer (5'-3')
OsWRKY24	GAATCTCAAAGACATGACACGACGG	CATCTGCATTCCTGGGCCCTC
OsCAT2	AGGAGGCAGAAGGCGACGATACA	TCTTCACATGCTTGGCTTCACGTT
OsJAZ9	GGCCGGTCGAGTTGGAA	GGTCAGGCTCGGCGAAAT
OsRR6	GTCCCCAACGTCAACATGATC	CACGTTCTCCGACGACATGAT
